# Study on the 4D printing performance of PFO occluder frames based on PLA/PEG filaments

**DOI:** 10.1080/15685551.2025.2602930

**Published:** 2025-12-16

**Authors:** Hai Ding, Shuwen He, Bingxin Ma, Fengjun Chen

**Affiliations:** School of Materials Science and Engineering, Henan Institute of Technology, Xinxiang, Henan, China

**Keywords:** PFO occluder, 4D-printed, PLA/PEG, orthogonal experiment, response surface methodology

## Abstract

This paper focuses on the study of factors influencing the performance of 4D-printed PFO occluder frames. First, polyethylene glycol (PEG, Mn = 2000) was used to blend and modify polylactic acid (PLA). At a PEG content of 15%, the glass transition temperature of the blend decreased from 74°C for pure PLA to 56°C, with a significant increase in hydrophilicity and the water contact angle decreased from 74° to 62°. Regarding mechanical properties, tensile strength decreased from 60.0 ± 1.8 MPa to 27.5 ± 1.2 MPa, while elongation at break increased substantially from 4.8 ± 0.8% to 300 ± 50.2%.When PEG content was increased to 25%, the glass transition temperature decreased to 58°C, hydrophilicity continued to enhance, and the contact angle decreased to 54°. At this point, tensile strength decreased to 15.6 ± 2.0 MPa, while elongation at break further increased to 432.5 ± 30.2%. Then, orthogonal + response surface experiments were conducted to optimize the FDM 4D printing process parameters, identifying fill rate as the key factor influencing the shape recovery rate and tensile strength of PLA parts. A regression model was established linking tensile strength to printing temperature, speed, and fill rate. Mechanical testing indicated that the 8-ligament occluder exhibited the best mechanical properties, while shape memory recovery rate tests showed that occluders made from PLA/PEG2K-25 filament had superior shape recovery rates compared to those made from PLA/PEG2K-15. The study demonstrates that through PEG modification of PLA and control of the 4D printing process, comprehensive optimization of the mechanical and shape memory properties of PFO occluder frameworks can be achieved.

## STATEMENT OF SIGNIFICANCE

This study proposes a strategy for preparing high-performance PFO occluder frames by comprehensively considering 4D-printed filament materials and printing process parameters. First, PLA is a biodegradable aliphatic polyester approved by the FDA for direct contact with biological fluids. However, its raw material has drawbacks such as high brittleness, low crystallinity, and low melt strength. Therefore, it is blended with polyethylene glycol (PEG, Mn = 2000) at different ratios to modify its properties and prepare 4D printing filament materials. Second, considering that process parameters such as fill rate, fill shape, printing temperature, and printing speed in FDM 4D printing contribute differently to various performance metrics (e.g. shape recovery rate and tensile strength), we employed orthogonal and response surface experimental design schemes to perform multi-objective optimization of 4D printing process parameters, with shape recovery rate and tensile strength as evaluation criteria. Finally, PFO occluders were 4D-printed using the prepared modified filament, and their performance was characterized. This study demonstrates that measures such as PEG-blended modification of PLA to prepare 4D printing filament and multi-objective optimization of 4D printing process parameters lay the foundation for the FDM 4D printing of high-performance PFO occluders.

## 1. Introduction

**Figure 1.**
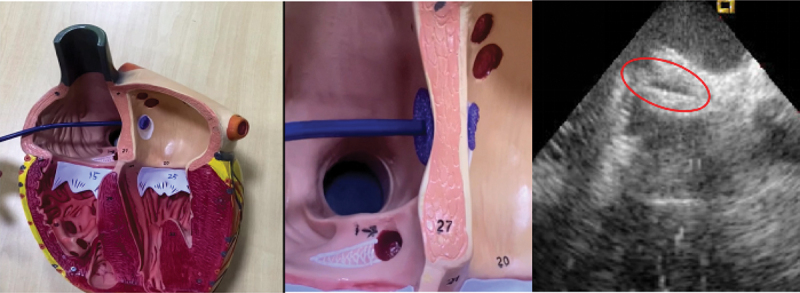
Working principle diagram of cardiac occluder.

**Figure 2.**
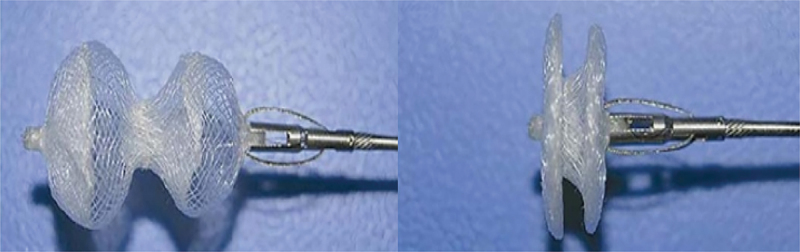
Fully degradable PLA occluder.

Congenital heart disease (CHD) represents a heterogeneous group of structural abnormalities of the cardiovascular system, arising from abnormal embryonic development. It is one of the most common congenital malformations, with a global incidence rate as high as 7–8 per 1000 [1]. The key medical device required for clinical treatment of this condition is a cardiac occluder. The delivery process involves a temporarily folded cardiac occluder being transported from outside the body into the body via a delivery device. Once it reaches the site of the cardiac defect, it unfolds into its permanent shape and is fixed in place to occlude the defect, ensuring unobstructed blood flow and improving cardiac function. An illustrative diagram of the occlusion mechanism of a cardiac occluder is shown in Figure 1 With the continuous development of materials science and medical devices, fully biodegradable cardiac occluders have become a research hotspot due to their ‘temporary support and gradual degradation’ characteristics [2,3]. These occluders can completely degrade and be absorbed by the human body after completing their therapeutic tasks, thereby avoiding the long-term side effects of traditional metal occluders. For example, the fully biodegradable occluder developed by Xianjian Technology Co., Ltd. (see Figure 2.) has a frame woven from PLA material and is covered with a layer of PLA barrier film. It can expand on its own, and one end of its locking device is connected to the left coil, while the other end is connected to the delivery system [4].

Building upon 3D printing technology, 4D printing refers to the process of manufacturing objects that can undergo changes in shape, properties, or functionality over time under the influence of external environmental factors through structural design and process optimization [5–7]. 4D printing enables the transformation of intricate structures within confined spaces, addressing the challenge of constructing precise internal human structures – a task that traditional manufacturing techniques struggle to achieve in the medical field [8]. With the emergence of 4D printing technology, FDM printing-based personalized medical applications have gained greater research value. Longinotti et al. [9] treated an infant with multiple complex muscular VSDs at the apex of the heart, who also had a large ASD leading to pulmonary hypertension. Conventional treatment methods were unable to satisfactorily resolve the issue, so the team performed palliative pulmonary artery banding. Two years later, they successfully closed the defect using an Amplatzer occluder via 3D printing technology, thereby removing the pulmonary artery band. Li et al. [10] studied 62 patients diagnosed with multiple ASDs (with defect distances greater than 7 mm) and found that the occluder sizes estimated using 4D-printed models were similar to those used during surgery. This demonstrates the significant clinical application value of 4D printing technology in the interventional treatment of complex congenital heart disease (CHD).

Polylactic acid (PLA) is a typical biodegradable polymer material that has been widely used in the medical device field due to its excellent biocompatibility and mechanical properties. However, PLA itself has high brittleness and uncontrollable degradation rates, limiting its application in complex cardiovascular interventional treatments [11]. Therefore, flexible polymer material polyethylene glycol (PEG) was first introduced to improve the flexibility and degradation behavior of PLA; then, orthogonal and response surface method experiments were conducted to study the influence of FDM 4D printing process parameters on the mechanical properties and recovery behavior of the components; finally, a PFO cardiac occluder frame based on modified PLA material was prepared using the 4D printing process, and its performance was studied and discussed.

## 2. Modification and characterization of 4D printing filaments

### 2.1. Preparation of 4D printing filament

Currently, biodegradable aliphatic polyesters available for constructing biodegradable cardiac implantable devices with different functions include PLA, poly (dioxanone) (PDO), poly(hydroxyacetic acid) (PGA), and polycaprolactone (PCL), among others, with their properties summarized in Table 1. Among these, PLA was approved by the U.S. Food and Drug Administration (FDA) in 1970 for direct contact with biological fluids. Due to the different chemical activities of the lactic acid and lactide used in the synthesis of PLA, three stereoisomers can be obtained: Poly(L-lactic acid) (PLLA), Poly(D-lactic acid) (PDLA), and racemic polylactic acid (PDLLA).

**Table 1.** Physical properties and degradation cycles of clinically used biodegradable polymers [12–15].

Polymer	Melting Point /(°C)	T_g_/(°C)	Tensile Strength/(MPa)	Modulus/(GPa)	Degradation Cycle/(Months)
PLLA	173~178	60~65	50~70	3.0~4.0	>24
PDO	110	−10~0	30~40	1.0~2.0	6~12
PCL	53~63	−65~-60	Approx.20	Approx.0.4	>24
PGA	225~230	35~40	Approx.115	Approx.7.0	6~12

#### 2.1.1. PLLA performance

**Figure 3.**
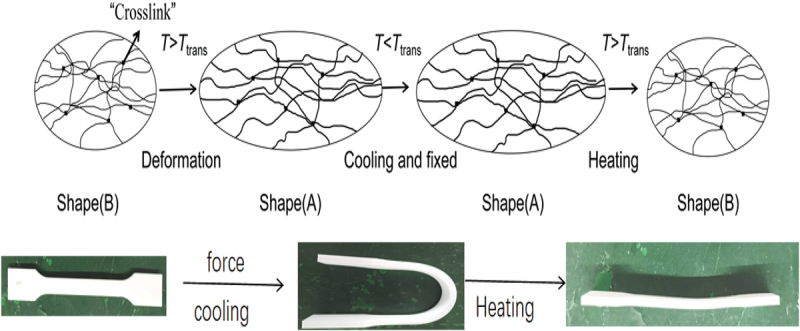
Schematic of shape memory behavior.

PLLA is an α-hydroxy acid polymer with L-lactic acid enantiomers, which has high tensile strength, low elongation at break, good biocompatibility, high tensile modulus of elasticity, and ease of processing, and in particular, it has shape-memory properties as shown in Figure 3. Under physiological conditions, PLLA is further degraded through the citric acid cycle and finally excreted as H_2_O and CO_2_.The degradation time of PLLA in vivo is about 2 ~ 3 years, which may lead to persistent local inflammatory reactions.The degradation time of PLLA is related to its molecular weight magnitude, crystallisation temperature, size and morphology as well as the external environment, so that the degradation rate of PLLA can be regulated by the degree of its crystallinity, porosity and molecular weight can be regulated to control it. Therefore, PLLA has become one of the most promising implantable materials compared to other biodegradable materials. However, the PLLA has the disadvantages of high brittleness, low crystallinity, and low strength of the melt [16,17]. In view of the inherent defects of the PLLA, its properties such as toughness, strength, and crystallinity need to be optimised through a variety of modification means to meet the practical application requirements, and thus to enhance the comprehensive performance of PLLA composites and to expand the application scenarios.(For ease of reading, PLA will be used throughout the rest of this document to refer to PLLA.)

#### 2.1.2. Blending modification of PLA by PEG

Currently, there are two methods for modifying PLA: copolymerization modification and blending modification. Copolymerization modification involves a complex process, is costly, and alters the inherent properties of PLA. In contrast, blending modification produces blends that retain both the renewable nature of the raw materials and the biodegradability of the final product, making it the preferred method [18,19]. During blending modification, the interfacial adhesion force between phases in the blend system formed by adding inorganic substances or macromolecular polymers to PLA is weak, resulting in limited modification effects. Therefore, small molecules such as PEG and PPG are often added to reduce the surface tension after mixing. PLA and PEG have very similar solubility parameters, theoretically exhibiting excellent compatibility [20].

PEG significantly enhances the properties of polylactic acid (PLA) in multiple aspects: In terms of mechanical properties, the addition of PEG markedly improves the material’s flexibility. PEG molecular chains intercalate between PLA chains, weakening intermolecular forces and increasing segmental mobility. This enables the blend to undergo plastic deformation rather than brittle fracture. Thermally, PEG introduction markedly lowers the material’s glass transition temperature (T_g_). Its plasticizing effect loosens PLA chain stacking, enabling rubber-like behavior at lower temperatures. Regarding biocompatibility and degradability, PEG itself exhibits excellent biocompatibility and suppresses non-specific protein adsorption on material surfaces. Simultaneously, the abundant hydrophilic groups in PEG chains – such as ether bonds (-O-) and terminal hydroxyl groups (−OH) – significantly enhance the hydrophilicity of PLA blends. Therefore, PEG is widely used to enhance the mechanical properties and degradability of PLA [21–24].

The melting point of PEG is directly proportional to the molecular weight, gradually approaching the limit of 67 °C. With the increase of PEG addition, the torque value of PLA/PEG blends decreases, and the extruder screw is prone to slip, resulting in inhomogeneous blending. In order to better characterize the effect of different relative molecular masses of PEG on the processing and plasticizing behaviour of the blended system, the addition of PEG should not be more than 30%, and therefore the PEG addition was set at: 15% and 25%. The materials used in this experiment: PLA with a relative molecular mass (Mn) of 120,000, Nature-works, U.S.A., and plasticiser: PEG (Mn = 2000, 15%, 25%). First, dry the PLA pellets in a vacuum oven at 60°C for 12 hours to remove moisture. Subsequently, melt-blend the PLA with PEG using a Haake Rheomix 600 mixer at 175°C and a main shaft speed of 40 rpm for 5 minutes to ensure thorough mixing. After blending, feed the composite material into a granulator for comminution and granulation, then air-cool it at room temperature for 24 hours. The cooled granules are then fed into the Nanjing Kebelong CTE35 twin-screw extruder for the purpose of blending filament. The extrusion parameters are set as follows: temperatures of 175°C, 180°C, 185°C and 180°C are used in sequence, with a main shaft speed of 40 rpm. The resulting filament diameter is measured at 1.75 mm, with a standard deviation of ±0.05 mm. Inspection of the surface revealed that it was smooth, hard in texture and transparent. The prepared PLA/PEG blends were named PLA/PEGx-y, with ×denoting the molecular weight of PEG and y denoting the mass fraction of PEG in the blend, notated as PLA/x-y. For example, PLA/2K-15 denotes the following meanings, the content of PLA is 95%, and the molecular weight of PEG is 2K with a mass fraction of 15% in the blend.

### 2.2. Material properties and characterisation

The equipment used in the characterization and analysis of material properties is shown in Table 2.

**Table 2.** Experimental instruments and sources.

Instrument	Model	Manufacturer
Electronic Balance	AL104	Mettler Toledo (Shanghai)
Vacuum Drying Oven	DZF-6020AB	Shanghai Yiheng Scientific
Synchronous Thermal Analyzer	STA449 F5	Netzsch (Germany)
Optical Microscope	SZM-7045	Ningbo Sunny Optical
Universal Testing Machine	GT100	NCS Testing Technology
Contact Angle Goniometer	SDC-100	Shending Precision Instruments

#### 2.2.1. Thermodynamic properties

**Figure 4.**
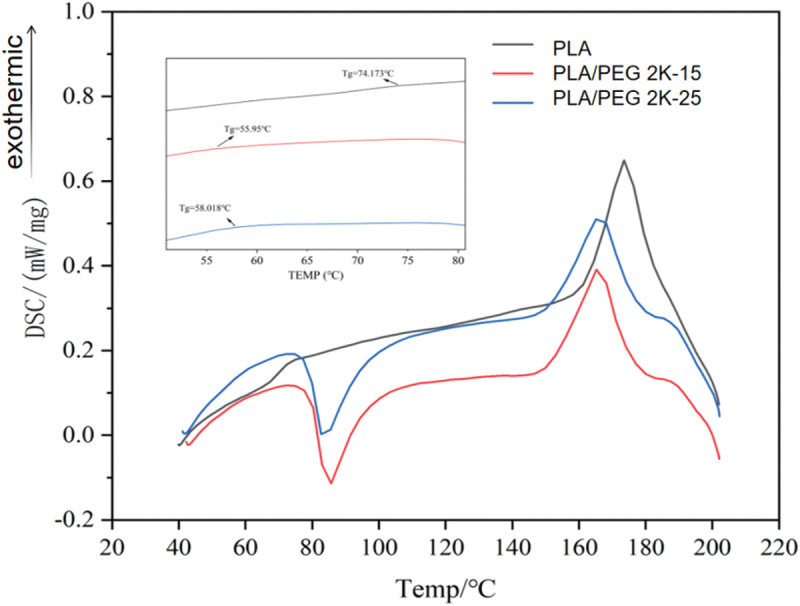
DSC curves of PLA/PEG blends.

**Figure 5.**
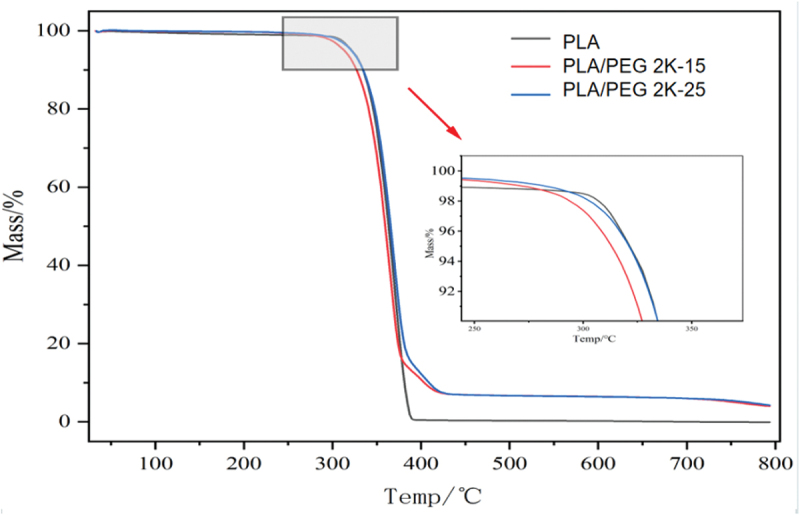
TGA curves of PLA/PEG blend.

A synchronous thermal analyser was used for the thermal analysis of the prepared modified materials, with N2 as the protective gas, the temperature was increased from room temperature to 220°C at a rate of 10°C/min, and held for 5 min in order to eliminate the effect of thermal history; then the temperature was reduced to 20°C at a rate of 20°C/min, and kept at a constant temperature for 5 min; and then finally, the temperature was increased to 220°C at a rate of 20°C/min, and kept at a constant temperature for 5 min. The results are shown in Figure 4. N_2_ as a protective gas, take 10 °C/min heating rate from the initial ambient temperature of 25 °C to 800 °C, record the weight changes in the sample testing process and heat flow rate data, the results are shown in Figure 5.

The phase transition behavior of PLA/PEG composites is closely related to the mass fraction of PEG. When the PEG content is 0, the glass transition temperature (T_g_) of pure PLA is 74.17 °C, with a melting enthalpy of 83.14 J/g. Upon introducing PEG, the Tg of the blends decreases significantly, with cold crystallization enthalpies (ΔH_cc_) of 18.84 J/g and 18.27 J/g, respectively. This is primarily attributed to the high mobility of PEG molecular chains, which weakens intermolecular interactions in PLA, enhances segmental mobility, thereby lowering Tg and altering cold crystallization behavior. Specifically, the cold crystallization peak temperatures (T_cc_) of PLA/PEG2K-15 and PLA/PEG2K-25 decreased to 85.71 °C and 82.69 °C, respectively. As PEG content increases, PLA’s Tcc continues to decrease. This phenomenon, along with the consistent glass transition temperature (T_g_), indicates good compatibility between the two within a certain range. PEG addition enhances the mobility and flexibility of PLA molecular chains, thereby promoting crystallization. Within an appropriate PEG addition range, increased molecular chain mobility corresponds to enhanced crystallinity. Pure PLA exhibits a melting peak at 173.47 °C, whereas the melting temperatures (T_m_) of PLA/PEG2K-15 and PLA/PEG2K-25 decrease to 168.15 °C and 165.18 °C, respectively. This indicates that PEG significantly lowers PLA’s melting point by weakening intermolecular forces, thereby affecting its thermal properties. Notably, PLA/PEG2K-25 and PLA/PEG2K-15 exhibited smaller changes in cold crystallization and melting peaks, suggesting that higher PEG content reduces compatibility with PLA, promoting phase separation and diminishing its modifying effect on PLA [25,26].

According to the data in Table 3, the initial thermal decomposition temperature (Tₒ_nset_) of pure PLA is 289.90 °C. After adding PEG with a molecular weight of 2000, the Tₒ_nset_ of the PLA/PEG2K-15 blend decreased to 287.35 °C, indicating reduced thermal stability. However, when the PEG content increased to 25%, Tₒ_nset_ recovered to 292.35 °C. This non-monotonic variation indicates that PEG introduction generally weakens the thermal stability of PLA/PEG blends. The mechanism involves PEG enhancing PLA molecular chain mobility by reducing segmental rotation barriers and expanding free volume, thereby promoting thermal decomposition. However, as PEG content further increases, its partial phase separation within the PLA matrix reduces compatibility, thereby mitigating PEG’s negative impact on material thermal stability. Numerous studies have reported an inverted U-shaped relationship between PEG content and PLA thermal stability, consistent with the trend observed in this study [27–29].

**Table 3.** TGA analysis.

Materials	T_onset_ (°C)	thermal weight loss5%/Temp (°C)	thermal weight loss10%/Temp (°C)
PLA	289.90	321.73	335.21
PLA/PEG-15	287.35	313.09	327.22
PLA/PEG-25	292.35	321.12	333.35

#### 2.2.2. Mechanical properties

**Figure 6.**
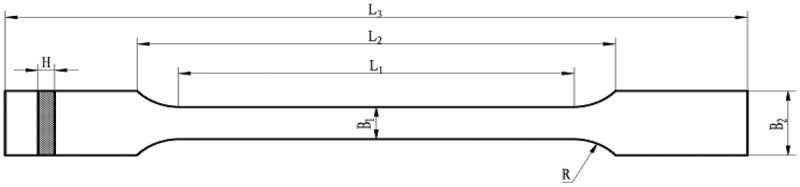
Specimen diagram.

Tensile specimens :L3 = 170 mm，L2 = 110 mm，L1 = 80 mm，R = 25 mm，B2 = 20 mm，B1 = 10 mm，H = 4 mm (See Figure 6)，were printed at 220°C with 0.1 mm layer thickness. In accordance with ASTM D638 testing standards, tensile property tests were conducted at room temperature (25°C) on FDM-printed dumbbell specimens using an universal testing machine (Model GNT100, capacity 100 kN). The tensile speed was set at 5 mm/min, with five replicate tests performed for each component material. The results are shown in Table 4.

**Table 4.** Mechanical properties of modified PLA.

Test No	PEG molecular weight	PEG content/(%)	Tensile strength/(MPa)	Elongation at break /(%)
1		0	60.0±1.8	4.8±0.8
2	2000	15	27.5±1.2	300±50.2
3	2000	25	15.6±2.0	432.5±30.2

Tensile test results indicate that when the PEG content reaches 15%, the tensile strength of the PLA/PEG2K-15 composite decreases to 27.5 MPa, while the elongation at break significantly increases to 300%. This phenomenon can be attributed to the addition of PEG with a molecular weight of 2000, which significantly lowered the T_g_ of the composite material (by approximately 55°C, see Table 5). This enhanced the mobility of PLA molecular chains, resulting in a decrease in tensile strength accompanied by an increase in elongation at break [30]. As PEG content further increases, the free volume between PLA molecules continues to expand, enhancing molecular chain flexibility. However, the uniformity of PEG dispersion within the PLA matrix decreases (as evidenced by a rebound in glass transition temperature observed in thermodynamic DSC experiments), leading to reduced compatibility.

**Table 5.** Thermal analysis data of PLA/PEG blends.

Sample	Glass transition temperature T_g_/ (°C)	Cold crystallisation temperature T_cc_/ (°C)	Melting temperature T_m_/ (°C)
PLA	74.17	–	173.47
PLA/PEG2K-15	55.95	85.71	168.15
PLA/PEG2K-25	58.01	82.69	165.18

#### 2.2.3. Hydrophilicity

The hydrophilicity of biodegradable polymers directly affects their degradation rate in aquatic environments.The static contact angle is an important indicator of the hydrophilicity/hydrophobicity of polymer materials, which reflects the strength of the instantaneous hydrophilicity of the material surface in contact with water. It is usually considered that the contact angle is less than 90° of the surface of the material is hydrophilic, and the smaller the angle is, the better the hydrophilicity of the material is. Three kinds of filaments, PLA, PLA/PEG-15 and PLA/PEG-25, were used to print out 0.1 mm-thick sheets; the sheets were laid flat and fixed on slides, and droplets were slowly deposited on the surface of the film with a microsyringe, and the side view of the droplets was photographed with an optical contact angle meter at high speed to ensure the clear contour; the static contact angles at three different points were calculated using the Image J software and the average values were obtained to analyse the hydrophilicity of the materials. The hydrophilicity of the material was analysed by taking the average value of the static contact angle at three different points.

**Figure 7.**
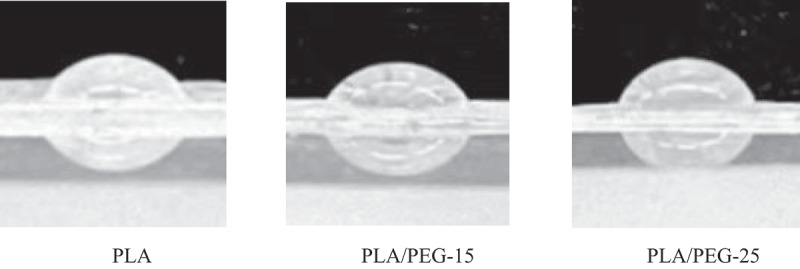
Contact angle diagrams of three filaments for printing thin films.

The average contact angles of the three materials were measured to be 73.445°, 62.758°, and 54.176°, respectively, See Figure 7. As the PEG content increases, the hydrophilicity of the copolymer material improves, which is consistent with the conclusions of Zhang et al. [31] regarding the hydrophilicity of PLLA/PEG blends. This is because PEG is a water-soluble polymer containing hydrophilic groups such as terminal hydroxyl and ether groups, which readily interact with polar water molecules. When PEG is incorporated into PLA, water molecules can more easily diffuse across the surface of the blend material, reducing the contact angle and enhancing hydrophilicity. As the PEG content increases, the material’s hydrophilicity also gradually improves.

## 3. Optimization of 4D printing parameters

The performance of PLA parts is closely related to their manufacturing process parameters, and changes in certain printing parameters can significantly affect the performance of PLA parts [32–37]. Jin Zhefeng [38] investigated the effects of FDM process parameters on the mechanical properties of PLA parts, Li Haimei [39] studied the influence of process parameters on the melt deposition dimensions of polylactic acid filament, Wen Zhou [40] examined the impact of fill patterns on bending modulus, and M. Eryildiz [41] optimized the shape memory effect of parts by adjusting sample thickness, nozzle temperature, deformation temperature, and holding time, while Zhao [42] investigated the effects of fill rate and model printing direction on the shape memory performance of PLA parts. While most of the aforementioned studies focused on mechanical properties, some also addressed shape memory performance and degradation functionality. However, comprehensive studies combining shape memory performance and mechanical properties are scarce. Therefore, this study takes PLA filament FDM printing parameters as the research object, with shape recovery rate and tensile strength as evaluation criteria. Using orthogonal experiments and response surface methodology to design the experimental scheme, the FDM printing performance of PLA filaments is investigated. The filament used in the experiment had a diameter of φ1.75 mm, a tensile strength of 55.7 MPa, and a tensile modulus of 3.0 GPa. The printing temperature range was 190–230 °C. Use the equipment listed in Table 6 to prepare the tensile test specimens shown in Figure 6 and characterize their performance.

**Table 6.** Experimental apparatus.

Equipment	Model	Manufacturer
FDM Printer	Bambu Lab A1	Shenzhen Bambu Lab
IR Drying Oven	SHD	Jiangmen Sante Instrument
Caliper	H1650-W	Anhui Measuring Tools
Universal Angle Gauge	613–01	Hagang Group
Stereomicroscope	SZM7045-B1	Shanghai Sunny Optical
Universal Tester	GNT 100	NCS Testing Technology

### 3.1. Shape memory performance

#### 3.1.1. Experimental design

Orthogonal experiment is a type of experimental design method used to study the effects of multiple factors and levels. To investigate the influence of factors such as fill rate, fill pattern, and printing direction on the shape recovery rate of the part [43,44], a three-factor, three-level orthogonal experimental design table was constructed，with an error column added to quantify the random fluctuations that cannot be avoided in the experiment, as shown in Table 7. A print head diameter of 0.2 mm, print speed of 90 mm/s, print temperature of 200 °C, print bed temperature of 65 °C, and layer thickness of 0.1 mm were selected, and the parts were printed according to the orthogonal experimental design.

**Table 7.** Table of test factor levels.

Level	Orientation(A)/	Infill Density(B)/ (%)	filler pattern(C)	Empty columns (D)
1	Horizontal	30	Honeycomb	1
2	Vertical	60	Grid	2
3	Lateral	90	Spiral	3

**Figure 8.**
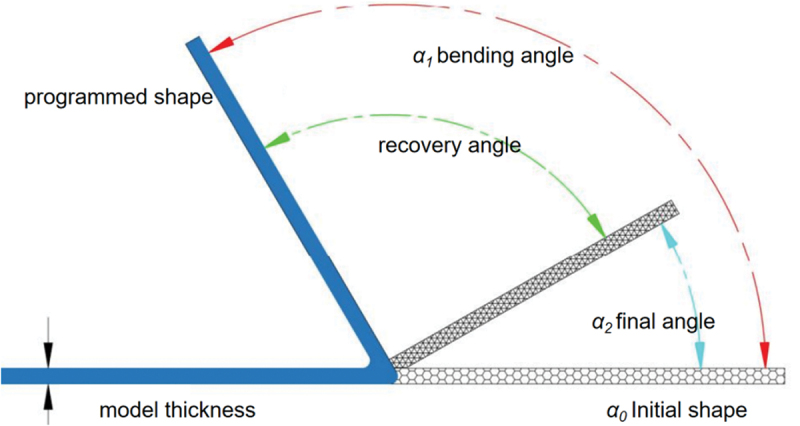
Schematic diagram of shape memory performance.

Measurement steps: Use the universal angle ruler to measure the initial angle of the specimen recorded as a_0_. Place the parts in the fixture and then put them into the water bath, set the temperature at 60°C, and hold them for two minutes after they are heated up to the set temperature; bend the parts to 90°and fix them for a certain period of time, and then put them into the 25°C water to cool down and set the shape quickly. Use the universal angle ruler to measure the bending angle as α_1_. again put the parts into the water bath at 60°C, hold the temperature for two minutes, take out the specimen and put it into cold water to set the shape quickly, and then measure the rebound angle α_2_, as shown in Figure 8. use formula 1 to calculate the shape recovery rate.

(1) $$\mathrm{RR}=\frac{\alpha 2-\alpha 1}{\alpha 0-\alpha 1}$$

Where $\alpha_{0}$、$\alpha_{1}$_、_$\alpha_{2}$ = initial, programmed, and recovered angles.

#### 3.1.2. Results & analysis

Five independent samples were prepared under each experimental condition, and the test results are shown in Table 8.

**Table 8.** Results of orthogonal experiments on shape recovery rate.

Test No	Factor				Shape recovery rate/(%)
	Print Direction(A)/	Filling Rate (B)/ (%)	Filling pattern (C)	Empty columns (D)	
1	1	1	1	1	93.14±1.48
2	1	2	2	2	91.38±1.52
3	1	3	3	3	83.52±1.65
4	2	1	2	3	85.82±1.59
5	2	2	3	1	89.58±1.51
6	2	3	1	2	82.69±1.68
7	3	1	3	2	88.62±1.55
8	3	2	1	1	91.45±1.49
9	3	3	2	3	75.09±1.72
K1	268.04	267.58	267.28	274.17	
K2	258.09	272.41	252.29	262.69	
K3	255.16	241.3	261.72	244.43	
k1	89.347	89.193	89.093	91.39	
k2	86.03	90.803	84.096	87.563	
k3	85.053	80.433	87.24	81.476	
R	4.30	10.37	4.99	1.63	

The statistical results in Table 8 indicate that, it can be found that B (filling rate) > C (filling pattern) > A (printing direction) > D (empty column), which indicates that the filling rate has the greatest influence on the shape recovery rate of PLA fabricated parts; and K2 > K1 > K3 of the filling rate factor, i.e., 60% of the filling rate is the optimal reference value, which is the same as that obtained by Villacres [45] in the study. The reason was analysed: moderate voids provide the necessary space and freedom for the PLA molecular chains to recover their orientation when subjected to heat. Too high a filling rate may limit shape recovery by increasing the rigidity of the part due to densification locking up the chain segments, while too low a filling rate may result in a lack of strength due to the high porosity providing too much free volume and thus making it difficult to recover efficiently after deformation. Honeycomb structures typically have superior in-plane stiffness and energy absorption compared to linear structures such as straight lines and meshes, and the design of their junctions may be more effective in storing elastic strain energy during deformation and more uniformly driving the recovery of the overall structure when subjected to heat. Horizontal printing exhibits superior shape memory performance in specimens, indicating that printing orientation is a key factor influencing this property. The fundamental reason lies in the fused deposition process, where the shear forces exerted by the nozzle extrusion combined with the rapid cooling effect after deposition induce high molecular chain orientation along the print path. This results in an anisotropic microstructure that provides a significant driving force for shape recovery. The smallest difference in the K values for the print direction makes it clear that the print direction has the least effect on the shape recovery performance. This indicates that the interlayer bond strength is not a major contradiction affecting shape memory recovery in the bending deformation mode. In summary, A1B2C1 is the optimal solution, i.e., horizontal printing, 60% filling rate, and honeycomb filling group is the optimal combination for this experiment. The ANOVA data are shown in Table 9, and checking the table shows that F_0_. _05_(2, 2) = 19.0, so the infill rate has a significant effect on the results, the printing direction and infill pattern did not reach a significant level; the mean square value of the empty column is 1.90, which suggests that there may be errors or secondary factors in the experiment.

**Table 9.** ANOVA of orthogonal experiments on shape recovery rate.

Source of Difference	Sum of Squares	Degree of Freedom	Mean Square	F-value	Significance
Print Orientation (A)	38.85	2	19.42	10.22	Not Significant
Filling Rate (B)	180.80	2	90.40	47.57	Significant
Filling Pattern (C)	39.20	2	19.60	10.31	Not Significant
Error	3.80	2	1.90		
Combined	262.64	8			

### 3.2. Mechanical performance experiment

#### 3.2.1. Mechanical properties experimental program

To further investigate the influence of FEM forming parameters and their interactions on the mechanical properties of parts in 4D printing processes, response surface experiments were designed based on the Box-Benhnken experimental principle [46–49]. Referring to the research findings on the mechanical properties of FDM printing [50,51], tensile strength was selected as the response value, and printing temperature, printing speed, and fill rate were chosen as the working parameters. An experimental factor table was constructed (Table 10). Finally, a multiple regression equation was established and optimized to determine the optimal printing parameter combination.

**Table 10.** Table of factor levels for response surface tests.

Level	Temp(A)/ (°C)	Speed(B)/ (mm/s)	Infill(C)/ (%)
−1	190	20	20
0	210	60	60
1	230	100	100

#### 3.2.2. Results & analysis

A three-factor, three-level Box-Behnken test with 15 groups was designed using Design-Expert 13.0 software. Under each set of experimental conditions (i.e., each combination of printing parameters), at least two independent build batches were conducted. Within each batch, three valid specimens (n = 3) were independently prepared and tested. Stereomicroscopic observation revealed that all PLA specimens exhibited typical brittle fracture characteristics, with macroscopically smooth fracture surfaces displaying pronounced mirror effects. These findings align with the research by A. R. Torabi et al [52]. However, distinct microstructural differences were observable across groups under varying printing parameters (including printing temperature, infill density, and printing speed). These differences manifested as variations in dimple distribution, filament orientation, and interlayer bonding. Such microstructural variations led to changes in macroscopic mechanical properties. The specific tensile strength test results are presented in Table 11.

**Table 11.** Experimental results.

Test No	Printing temperature (A)/(°C)	Printing speed (B)/(mm/s)	Filling rate (C)/(%)	Tensile strength/(Mpa)
1	190	20	60	45.7±1.3
2	230	20	60	43.0±1.3
3	190	100	60	39.6±1.4
4	230	100	60	48.9±1.4
5	190	60	20	34.9±1.1
6	230	60	20	37.8±1.0
7	190	60	100	46.9±1.3
8	230	60	100	47.3±1.2
9	210	20	20	39.5±1.2
10	210	100	20	41.1±1.2
11	210	20	100	46.6±1.4
12	210	100	100	46.4±1.4
13	210	60	60	45.6±1.3
14	210	60	60	46.1±1.3
15	210	60	60	45.8±1.3

Despite normal experimental variations, all repeated tests consistently demonstrate that printing parameters – particularly infill density, printing temperature, and printing speed – significantly influence the mechanical properties of printed parts. The magnitude of each parameter’s effect exhibits stable, repeatable trends rather than random errors.The multiple regression equations of tensile strength (Y) and printing temperature (A), printing speed (B) and filling rate (C) were constructed without model misfit:

(2) Y=45.57+1.28A+0.65B+3.98C−1.48AB−0.73AC+0.23BC−0.94A−1.94B−1.71C

To test the significance of the quadratic polynomial regression model, analysis of variance (ANOVA) and significance test were performed. The results are shown in Table 12. The ANOVA shows that the regression model has an F-value of 8.75 and a probability of significance of P = 0.0081 < 0.01, which indicates that the model is extremely statistically significant; the out-of-fit term test of P = 0.2076 > 0.05 indicates that the model is statistically valid; and the value of the pure error is 0.86, which indicates that there is a high degree of experimental precision. The coefficient of determination R^2^ of the regression model is 0.9183 which indicates that the model has excellent predictive power.

**Table 12.** Table of analysis of variance on damage rate.

Source	Sum of squares	Degree of freedom	Mean square	F-value	P-value	Significance
Model	212.94	9	23.66	8.75	0.0081	*
A	19.66	1	19.66	7.27	0.0311	*
B	5.06	1	2.21	0.86	0.3782	
C	190.32	1	190.32	70.38	<0.0001	*
AB	17.47	1	17.47	6.46	0.0382	*
AC	4.28	1	4.28	1.58	0.2478	
BC	0.42	1	0.42	0.16	0.7043	
A2	7.92	1	7.92	2.93	0.1305	
B2	33.69	1	33.69	12.46	0.0094	*
C2	26.18	1	26.18	9.68	0.0169	*
Residuals	17.21	7	2.47			
Loss of fit term	15.58	5	3.44	3.44	0.2076	
Pure error	1.72	2	0.86			
Total deviation	231.87	16				

The p-value for fill rate (C) is < 0.0001, indicating it is a highly significant factor; significant factors include the interaction between print temperature and print speed (AB), the speed squared term (B^2^), and the fill rate squared term (C^2^). This indicates that the tensile strength of PLA material 4D printed parts is primarily influenced by the fill rate. Additionally, there is a significant interaction between print temperature and print speed, and optimizing their combination can significantly enhance tensile strength.

#### 3.2.3. Interaction effects of factors

**Figure 9.**
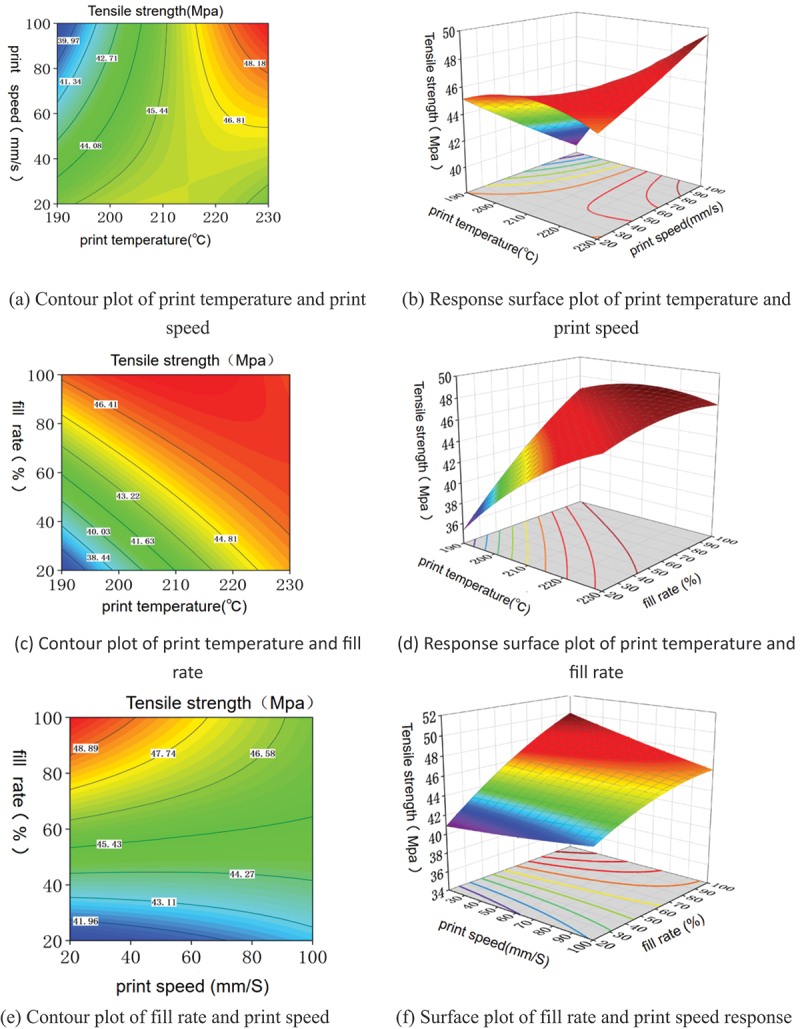
Factor interaction plots.

The contour and response surface plots are drawn according to the experimental data, as shown in Figure 9. In Figure 9(a), the contour lines formed by printing temperature and printing speed show an oblique ellipse shape, indicating that there is an interaction between the two factors; in Figure 9(b), the surface formed by printing temperature and printing speed shows a steeper bending state, indicating that the interaction of the two factors has a more prominent effect on the tensile strength, which is in line with the results in Figure 9(a). The essence lies in the correlation between the melt rheological behaviour and the cooling rate, from the molecular orientation of PLA materials, crystallization: low temperature + low-speed case chain segment relaxation is sufficient, the molecular chain is fully diffused leading to the interlayer entanglement enhancement, so the tensile strength is increased; low temperature + high-speed case chain segment freezing is too fast, the molecular chain is not sufficiently diffused leading to the interlayer weak bonding and thus a decrease in the tensile strength; high temperature + low-speed case chain segment overly In the case of high temperature and low speed, the chain segments are too relaxed, and the molecular chains are easy to be untangled, so the crystallinity decreases, which leads to the decrease of tensile strength; in the case of high temperature and high speed, the shear induces the molecules to undergo orientation, forming a self-reinforcing structure, therefore, the tensile strength is improved. From the PLA material viscosity and shear rate: low temperature + high-speed case, the melt viscosity is larger extrusion easy to expand resulting in interlayer porosity rise, thus in the tensile experiment is prone to stress concentration, so the tensile strength decline; high temperature + high-speed case, the melt viscosity drop high-speed extrusion melt uniformity can be improved, so its tensile strength rose.SEM images of the printed specimens reveal numerous pores on the cross-section, appearing as rhombic or triangular shapes. These primarily originate from micro-defects formed by incomplete interlayer fusion. Weak bond zones and interfilament gaps act as stress concentration points, significantly impacting the tensile strength of the fabricated parts. Using ImageJ image processing software to analyze the porosity of fracture surface photographs obtained from scanning electron microscopy (SEM), the porosity ranged from 4.2% to 2.8%. Reducing the porosity of the final product is crucial for enhancing interfacial bonding strength and improving the mechanical properties of 3D-printed parts. Adding highly fluid oligomers can promote neck growth at the filament interface, increase molecular chain entanglement at the interface, thereby reducing porosity and enhancing the mechanical strength of the printed product [53]. From Figure 9(d) can be seen under the interaction of filling rate and printing temperature, higher filling rate can increase the overall tensile strength of the parts, but too high or too low printing temperature will lead to a decrease in the tensile strength of the parts, the reason for this is that PLA belongs to the semi-crystalline materials and its mechanical properties are affected by the molecular chain crystallisation and orientation of the impact of the critical threshold value. Therefore, there is a set of optimal 4D printing parameters for the mechanical properties of the printed parts.

#### 3.2.4. Optimal solution analysis

The optimal solution can be obtained from the quadratic polynomial regression equation of tensile strength fitted by Design-Expert: tensile strength of 49.2 MPa at a printing temperature of 222 °C, a printing speed of 47 mm/s, and a filling rate of 84%, and the average value of three experiments is 49.5 MPa under the same process conditions. Possible reasons for this are as follows: under the 222 °C printing temperature Under the medium shear speed, the PLA molecular chain obtains a higher degree of orientation along the extrusion direction; the crystallinity of the molecular chain is increased, and the synergistic effect of temperature and speed, i.e., melt rheology and thermodynamics synergistically leads to the uniform distribution of pores, and the moderate filler rate results in the uniformity of grain size; meanwhile, the appropriate porosity provides a free-volume cushioning effect on the expansion of cracks, and thus its tensile strength can be improved. The tensile strength is enhanced.

## 4. D-Printed PFO framework & performance

### 4.1. PFO occluder frame structure

**Figure 10.**
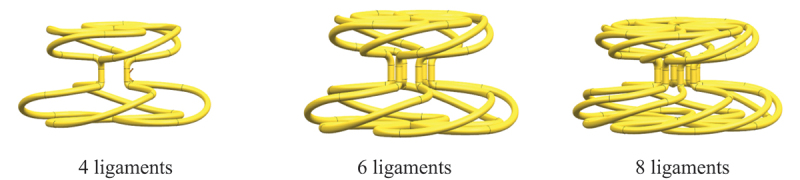
PFO models for different ligament models.

Many flexible biological tissues exhibit a nonlinear ‘J-shaped’ stress–strain curve, indicating significant extensibility and relatively high tensile strength at low stiffness [51,54,55]. Wave-shaped structures exhibit unique functional advantages in terms of motion flexibility, load-bearing capacity, and energy absorption. Therefore, a multi-ligament rotating wave structure was adopted in the construction of the PFO framework model to reduce the impact effects of human biomechanics. Parametric modeling was performed using three-dimensional modeling software to construct a PFO occluder framework model with a waist diameter of 8 mm, a large disk diameter of 40 mm, and a small disk diameter of 32 mm. In practical applications, factors such as the requirements for device delivery within the body (delivery difficulty, potential harm to the patient) and the load on the cardiac defect site must be comprehensively considered when selecting the number of ligaments. Therefore, four-, six-, and eight-ligament PFO occluder frame models were constructed for comparative experimental studies, as shown in Figure 10.

### 4.2. PFO frame 4D print moulding

**Figure 11.**
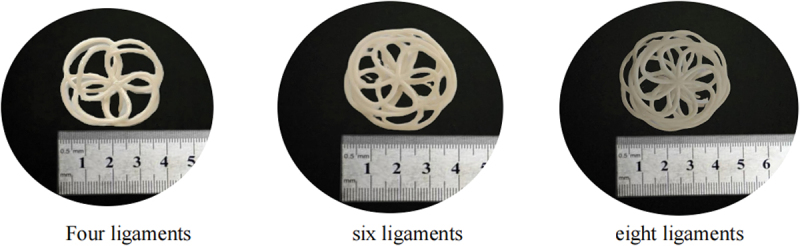
PFO frameworks with varying ligaments.

FDM 4D-printed parts exhibit anisotropy, with the tensile strength in the XY direction (within layers) being five times that of the Z-axis direction (between layers) [56,57]. Since the force direction of cardiac occluders primarily involves stretching or compression along the Z-axis, and considering that the shape memory effect was optimal when the part was placed horizontally in the previous shape memory performance experiments, the final printing orientation was determined to be horizontal. The PFO frame model was imported into slicing software for slicing. The 4D printing filament prepared in the previous section was selected, and the 4D printing parameters were set as follows: temperature 222°C, speed 47 mm/s, fill rate 85%, heated bed temperature 45°C, layer thickness 0.10 mm. The printed part is shown in Figure 11.

### 4.3. Characterisation of structural properties and functional evaluation of PFO scaffolds

#### 4.3.1. Mechanical properties

**Figure 12.**
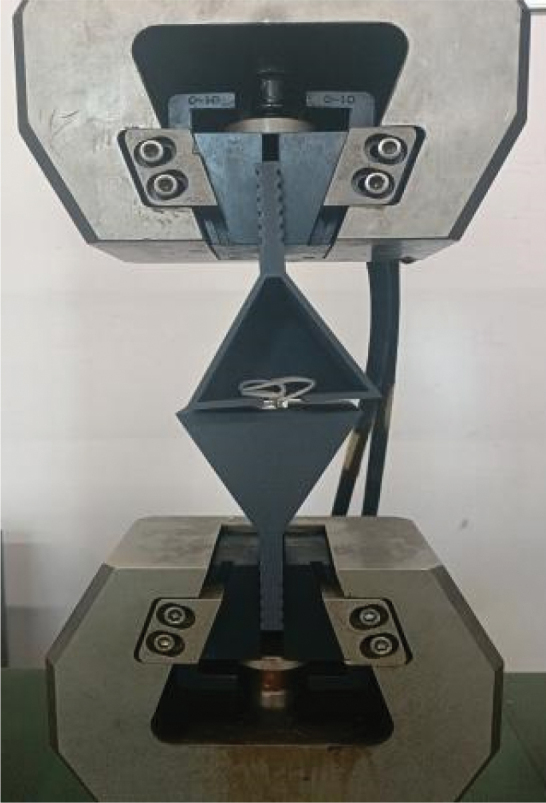
The tensile diagram of the PFO frame.

The PFO occluder is positioned at the waist, so a custom fixture was designed and the PFO occluder was placed between the upper and lower parts of the fixture for uniaxial tensile testing to evaluate the waist load-bearing capacity of the occluder. The test temperature was 30°C, and the loading rate was 2 mm/min. There is an 8 mm diameter hole in the middle of the fixture to simulate the clamping of the PFO occluder. As shown in Figure 12, the tensile diagram of the PFO frame.

During compression, occluders with different numbers of ligaments: (d) PLA, (e) 15% mPLA, (f) 25% mPLA.

**Figure 13.**
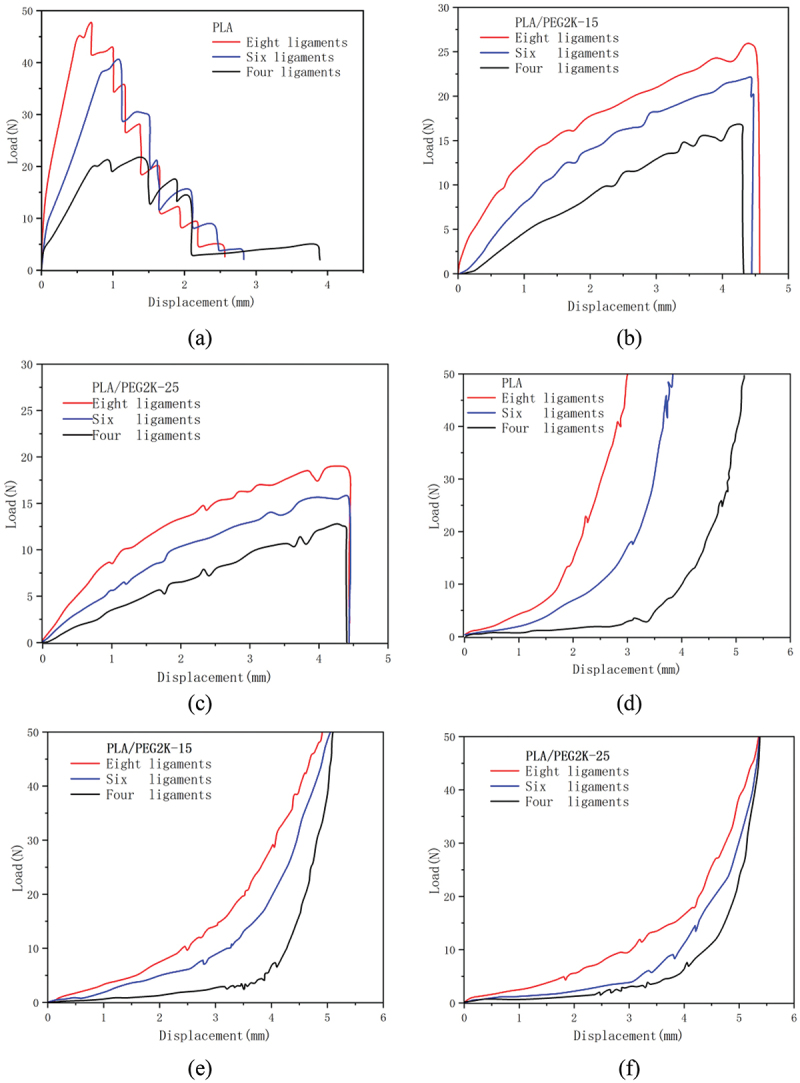
Load–displacement relationship curves of printed occluders.

Note: During tension, occluders with different numbers of ligaments: (a) PLA, (b) 15% mPLA, (c) 25% mPLA.

Figure 13(a-c) shows the uniaxial tensile load–displacement curves of shape memory occluders. The stepped curve in Figure 13(a) is related to the structure of the occluder; when the tensile force applied to the occluder reaches a certain value, the ligaments break sequentially; Among occluders made from pure PLA filament, the eight-ligament occluder has the highest load-bearing capacity. The load-bearing capacity of the six-ligament occluder is not significantly different from that of the eight-ligament occluder, but there is a significant difference between the six-ligament occluder and the four-ligament occluder. The maximum load capacity of the eight-ligament occluder is approximately 46 N, that of the six-ligament occluder is approximately 39 N, and that of the four-ligament occluder is approximately 22 N. Figure 13(b-c) shows occluders prepared from PLA/PEG2K-15 and PLA/PEG2K-25 filaments. Due to the significantly increased elasticity of the PLA/PEG-modified filaments, the occluders detached from the fixtures when subjected to a certain force during stretching, rather than being pulled apart. Among occluders made from the same filament material, the eight-ligament occluder exhibited the best load-bearing capacity. The maximum load-bearing capacity of the eight-ligament occluder made from PLA/PEG2K-15 filament material was 26 N, while that of the eight-ligament occluder made from PLA/PEG2K-15 filament material was 18 N.

To assess the ability of the PFO occluder to resist compressive loads after implantation, compressive testing was conducted on the PFO occluder, with the maximum compressive load set at 50 N. As shown in Figure 13(d-f), the compression load-displacement curve can be broadly divided into two sections. The first section is the ‘elastic zone,’ and the second section is the ‘dense zone,’ where the slope of the curve increases significantly. The eight-ligament occluder has the highest structural equivalent stiffness, the six-ligament occluder exhibits better toughness, and the four-ligament occluder has the poorest structural stability. As the PEG content increases, the ‘dense zone’ range of the occluder after compressive load gradually decreases, demonstrating good compressibility. Due to the occluder’s unique structure, it remains intact after a 50 N compressive load and exhibits excellent self-recovery capability.

#### 4.3.2. Shape memory performance

Shape Recovery Rate (SRR) is a quantitative measure (%) of the ability of a shape memory material to recover from a deformed state to its initial state, and the shape recovery time is usually measured in seconds (s). This experiment was conducted at room temperature (25°C). First, the sample was placed in a 55°C constant-temperature water bath for 3 minutes to complete shape-fixing programming, then transferred to room temperature (25°C) for cooling and setting. Subsequently, the sample was re-immersed in the 55°C constant-temperature water bath for 3 minutes to trigger shape recovery, and finally cooled at room temperature for shape measurement and data recording.

(3) $$\mathrm{SRR}=\frac{D_{3}-D_{2}}{{D_{1}-D}_{2}}\times 100\%$$

Description:D1 initial state; D2 programming state; D3 working state.

The shape recovery rates of occluder‘s frames made from different materials were all above 85%, with an average recovery rate exceeding 90%, indicating that the plugs have excellent shape recovery capabilities, as shown in Tables 13 and 14. The recovery rates of the upper and lower plates of the plugs were both lower than those in the height direction, which may be attributed to anisotropy caused by the printing direction. The average recovery rate of the occluders in the PLA/PEG2K-25 group was superior to that of the PLA/PEG2K-15 group, but the recovery time of the occluders in the PLA/PEG2K-15 group was shorter than that of the PLA/PEG2K-25 group. In terms of the recovery time for the shape of the occluders across all groups: 4 ligaments < 6 ligaments < 8 ligaments.

**Table 13.** PLA/PEG2K-15 shape memory performance.

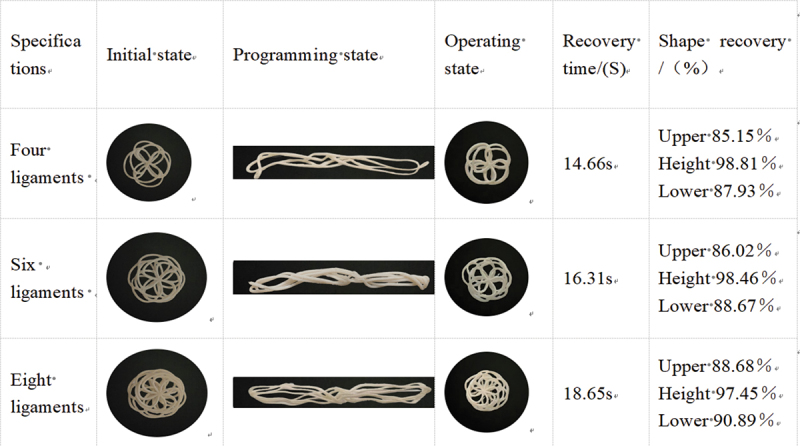

**Table 14.** PLA/PEG2K-25 shape memory performance.

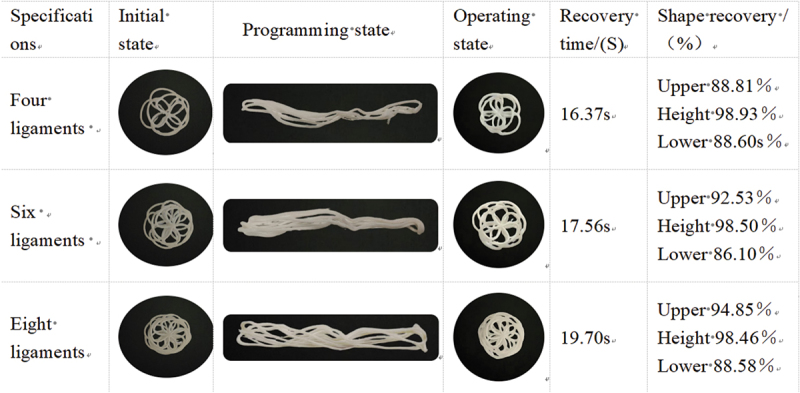

## 5. Conclusions and outlook

### 5.1. Main research conclusions

Material modification is effective: PLA was modified with PEG (molecular weight 2000) to enhance toughness. The glass transition temperatures of PLA, PLA/PEG2K-15, and PLA/PEG2K-25 were approximately 74°C, 56°C, and 58°C, respectively, with elongation at break of 4.8%, 300%, and 432%, respectively. tensile strength of 60 MPa, 27.5 MPa, and 15.6 MPa, respectively, and water contact angles of 73°, 62°, and 54°, respectively. The results indicate that by adjusting the molecular weight and addition amount of PEG, the shape transformation temperature, mechanical properties, and degradation rate of PLA/PEG blend 4D printing filaments can be comprehensively controlled.

Printing process optimization: Orthogonal experiments indicate that the factors affecting the shape recovery rate of PLA are ranked in order of importance as follows: fill rate > fill pattern > printing direction. The optimal combination is a fill rate of 60%, honeycomb fill pattern, and horizontal printing. Response surface analysis indicates that the factors influencing tensile strength, in order of importance, are: fill rate > printing temperature > printing speed. The optimal combination is a fill rate of 84%, printing temperature of 222 °C, and printing speed of 47 mm/s, with a significant interaction between printing temperature and printing speed. During 4D printing, the optimal 4D printing parameter combination can be determined by weighting the comprehensive requirements of shape recovery rate and mechanical properties.

Structural design feasibility: The four-, six-, and eight-ligament PFO models designed based on wave biomimetic principles can achieve shape transformation under external stimuli through 4D printing technology. PFO frame performance: Multi-ligament PFO occluder frames prepared from PLA, PLA/PEG2K-15, and PLA/PEG2K-25 filaments all exhibit good tensile and compressive strength, with an average shape recovery rate exceeding 90%. The average recovery rate of the occluder in the PLA/PEG2K-25 group was superior to that of the PLA/PEG2K-15 group, but the recovery time of the occluder in the PLA/PEG2K-15 group was shorter than that of the PLA/PEG2K-25 group. 4D-printed PFO occluder frames can meet the performance requirements for PFO frames.

### 5.2. Future directions

At present, the mechanical properties of degradable polymers fail to meet the higher level of mechanical properties of cardiac occluders under a variety of conditions of use such as strong support and fixation, soft tissue replacement and interventional delivery, which has become a key factor restricting the construction of cardiac occluders with degradable polymers. The degradation rate of degradable polymers or the growth rate of cardiac regenerative tissue cannot be matched exactly with the individual patient’s physique, and either too slow or too fast degradation will lead to the risk of human complications or implanted device failure. Regulating the degradation rate of biodegradable polymers to align with the tissue repair and regeneration speeds of patients with different body types, as well as the preparation of heterogeneous structures involving multi-material mixtures of varying sizes, presents a significant challenge in the development of biodegradable polymers [58,59]. Based on patient imaging data, selecting appropriate biodegradable polymer materials and optimizing 4D printing process parameters to prepare high-performance occluders, thereby establishing a ‘4D printing+’ technology system, is another major challenge in the development of high-performance occluders.

## Data Availability

The data that support the findings of this study are available from the corresponding author [Hai Ding], upon reasonable request. https://doi.org/10.6084/m9.figshare.29804627.

## References

[1] Goldstein SA, Krasuski RA. Complex congenital heart disease in the adult. Annu Rev Med. 2024;75(1):493–512. doi: 10.1146/annurev-med-050922-052324 38285514

[2] Yiming Y, Wenbin O, Fengwen Z, et al. Current status and prospects of interventional treatment for congenital heart disease in China. Chin J Thorac Cardiovasc Surg. 2022;29(10):1243–1253.

[3] Fengwen Z, Yi S, Xiangbin P, et al. Two cases of membranous ventricular septal defect treated with fully biodegradable occluders. Chin J Thorac Cardiovasc Surg. 2018;25(7):636–638.

[4] Li Y, Xie Y, Li B, et al. Initial clinical experience with the biodegradable AbsnowTM device for percutaneous closure of atrial septal defect: a 3-year follow-up. J Interv Cardiol. 2021:1–10. doi: 10.1155/2021/6369493 PMC8349294 34393667

[5] Mirasadi K, Yousefi MA, Jin L, et al. 4D printing of magnetically responsive shape memory polymers: toward sustainable solutions in soft robotics, wearables, and biomedical devices. Adv Sci. 2025;e13091:9–30. doi: 10.1002/advs.202513091 PMC13042656 40847440

[6] Ali SJA, Rahmatabadi D, Baghani M, et al. Design, processing, 3D/4D printing, and characterization of the novel PETG–PBAT blends. J Mater Sci. 2024;59(20):9150–9164. doi: 10.1007/s10853-024-09761-8

[7] Yarali E, Mirzaali MJ, Ghalayaniesfahani A, et al. 4D printing for biomedical applications. Adv Mater. 2024;36(31):2402301. doi: 10.1002/adma.202402301 38580291

[8] Lorenzo L, Biagio C, Francesco B, et al. Three-dimensional printing for hybrid closure of complex muscular ventricular septal defects. Ann Thorac Surg. 2021;113(2):e129–e132. doi: 10.1016/j.athoracsur.2021.04.049 33957097

[9] Li P, Fang F, Qiu X, et al. Personalized three-dimensional printing and echoguided procedure facilitate single device closure for multiple atrial septal defects. J Interv Cardiol. 2020;6:1–8. doi: 10.1155/2020/1751025 PMC7201835 32410914

[10] Tsai YA, Li T, Torres-Fernández LA, et al. Ultra-thin porous PDLLA films promote generation, maintenance, and viability of stem cell spheroids. Front Bioeng Biotechnol. 2021;9:674384. doi: 10.3389/fbioe.2021.674384 34195179 PMC8236593

[11] Malikmammadov E, Tanir TE, Kiziltay A, et al. PCL and PCL-based materials in biomedical applications. J Biomater Sci(Polym Ed). 2018;29(7/9):863. 29053081 10.1080/09205063.2017.1394711

[12] Middleton JC, Tipton AJ. Synthetic biodegradable polymers as orthopedic devices. Biomaterials. 2000;21(23):2335. doi: 10.1016/S0142-9612(00)00101-0 11055281

[13] Sabino MA, González S, Márquez L, et al. Study of the hydrolytic degradation of polydioxanone PPDX. Polym Degrad Stab. 2000;69(2):209. doi: 10.1016/S0141-3910(00)00062-8

[14] Park JM, Kim DS, Kim SR. Nondestructive evaluation of interfacial damage properties for plasma-treated biodegradable poly(p-dioxanone) fiber/poly(l-lactide) composites by micromechanical test and surface wettability. Compos Sci Technol. 2004;64(6):847. doi: 10.1016/j.compscitech.2003.09.009

[15] Yunbing W, Gaoyang G. Application of biodegradable polymer materials in cardiovascular treatment implants and interventional devices. J Tongji Univ (Nat Sci Ed). 2023;51(11):1649–1656.

[16] Song M, Li S, Zhu G, et al. Compatibilised and toughened of PLA/PCL blends via modified-chitosan linking amorphous regions: 4D printing and shape memory processes. Polym Test. 2023;125:108105. doi: 10.1016/j.polymertesting.2023.108105

[17] Suhua X, Jingxian Z, Wenhua Z. Preparation and mechanical properties of polylactic acid/nano-TiO_2_ composite materials for 3D printing. J Plast Eng. 2017;24(3):219–224.

[18] Shu G, Tao Z, Jin Z, et al. Study on the properties of PEG and PPG modified PLA materials. China Plastics. 2018;32(3):44–50.

[19] Jian X, Xuanbo W, Xinbo L, et al. Structure and cooling effect of poly(lactic acid)/poly(ethylene glycol) phase change materials. Plastics Ind. 2024;52(8):115–122.

[20] Lai WC, Liau WB, Lin TT. The effect of end groups of PEG on the crystallization behavior of binary crystalline polymer blends PEG/PLLA. Polymer. 2004;45(9):3073–3080. doi: 10.1016/j.polymer.2004.03.003

[21] Sringam J, Kajornprai T, Trongsatitkul T, et al. Shape memory performance and microstructural evolution in PLA/PEG blends: role of plasticizer content and molecular weight. Polymers. 2025;17(2):225. doi: 10.3390/polym17020225 39861296 PMC11768420

[22] Gao H, Xu S, Ai Y, et al. Effects of polyethylene glycol (PEG) on the structure and properties of flexible PEG/PLA composite films. J Appl Polym Sci. 2025;142(43). doi: 10.1002/app.57671

[23] Hu B, Chen Y, Zhai M, et al. Development of shape memory PLA with controllable stimulation temperature and dual-responsive properties by adding PEG. Iran Polym J. 2025;34(12):2091–2103. doi: 10.1007/s13726-025-01499-7

[24] Terzopoulou Z, Zamboulis A, Bikiaris ND, et al. A decade of innovation: synthesis, properties and applications of PLA copolymers. Prog Polym Sci. 2025;167:101991. doi: 10.1016/j.progpolymsci.2025.101991

[25] Mengyao S, Cao Y, Shike Z, et al. Recent advances in the study of poly(lactic acid) reinforcement and toughening. Polym Mater Sci Eng. 2022;38(3):183–190.

[26] Jianwei G. Study on the properties and plasticization mechanism of PLA/PEG composite materials. University of Science and Technology of China; 2021. doi: 10.27517/d.cnki.gzkju.2021.000681

[27] Cheng L. Design and mechanical properties of 4D-printed shape-memory polymer tissue engineering scaffolds. Harbin Institute of Technology; 2022.

[28] Yifan J. Preparation, structure, and properties of MRI-compatible polymer composite fibers. South China University of Technology; 2023. doi: 10.27151/d.cnki.ghnlu.2023.005033

[29] Mengmeng X, Fanwen Y, Han W, et al. Structure and properties of a ternary blending system of PLA/plasticizer/carbon nanotubes. Aging Application Synth Mater. 2017;46(5):6–9,18.

[30] Kuangsheng Z, Meirong T, Xiaojia X, et al. Crystallization and degradation behavior of PLA/PEG blends. J Chem Eng. 2021;72(2):1181–1190.

[31] Ansaripour A, Heidari-Rarani M, Mahshid R, et al. Influence of extrusion 4D printing parameters on the thermal shape-morphing behaviors of polylactic acid (PLA). Int J Adv Manuf Technol. 2024;132(3–4):1827–1842. doi: 10.1007/s00170-024-13470-6

[32] Elloumi A, Jerbi A, Ben Amor R, et al. A comparative study of gray relational analysis and VlseKriterijumska optimizacija I kompromisno resenje approaches for enhancing mechanical properties and productivity in 3D-printed copper-filled PLA parts. J Elastomers Plastics. 2024;56(5):675–692. doi: 10.1177/00952443241254939

[33] Rahmatabadi D, Khajepour M, Bayati A, et al. Advancing sustainable shape memory polymers through 4D printing of polylactic acid-polybutylene adipate terephthalate blends. Eur Polym J. 2024;216:13. doi: 10.1016/j.eurpolymj.2024.113289

[34] Li Z, Kong P, Liu X, et al. A fully biodegradable polydioxanone occluder for ventricle septal defect closure. Bioact Mater. 2023;24:252–262. 36632501 10.1016/j.bioactmat.2022.12.018 PMC9813538

[35] Agaliotis ME, Ake-Concha DB, May-Pat A, et al. Tensile behavior of 3D printed polylactic acid (PLA) based composites reinforced with natural fiber. Polymers. 2022;14(19):3976–3976. doi: 10.3390/polym14193976 36235924 PMC9570513

[36] Hasan RM, Davies JI, Paramanik A, et al. Fabrication and characterisation of sustainable 3d-printed parts using post-consumer PLA plastic and virgin PLA blends. Processes. 2024;12(4):760. doi: 10.3390/pr12040760

[37] Ben Amor R, Souissi S, Elloumi A, et al. Parametric optimization and modeling of the fused filament fabrication (FFF) manufacturing using recycled polyethylene terephthalate (PET) from water bottles. J Elastomers Plastics. 2025;57(6):1050–1071. doi: 10.1177/00952443251343320

[38] Ziyang W, Yifei C, Haimei L. Influence of process parameters on the melt deposition dimensions of polylactic acid filaments. Polym Mater Sci Eng. 2024;40(4):104–110.

[39] Zhou W, Meigui X, Xuhong W. Process optimization of PLA products via fused deposition modeling using the Taguchi experimental method. Eng Plastics Appl. 2021;49(1):58–62.

[40] Eryildiz M. Influence of process parameters on the shape recovery properties of 4d-printed polylactic acid parts produced by fused deposition modeling. J Materi Eng Perform. 2023;32(9):4258–4269. doi: 10.1007/s11665-023-07946-x

[41] Zhao W, Liu L, Zhang F, et al. Shape memory polymers and their composites in biomedical applications. Mater Sci Eng C-Mater Biol Appl. 2019;97:864–883. doi: 10.1016/j.msec.2018.12.054 30678978

[42] Liu K, Yang F, Wang X, et al. Influence of printing parameters and hinge structure on shape memory performance at the hinge in 4D origami structures. Smart Mater Struct. 2024;33(5):055045. doi: 10.1088/1361-665X/ad3ecb

[43] Kostopoulos G, Stamoulis K, Lappas V, et al. Shape morphing of 4d-printed polylactic acid structures under thermal stimuli: an experimental and finite element analysis. Aerospace. 2024;11(2):134. doi: 10.3390/aerospace11020134

[44] Villacres J, Nobes D, Ayranci C. Additive manufacturing of shape memory polymerseffects of print orientation and infill percent‐age on shape memory recovery properties. Rapid Prototyping J. 2020;26(9):1593‒1602.

[45] Hosseinzadeh M, Ghoreishi M, Narooei K. 4D printing of shape memory polylactic acid beams: an experimental investigation into fdm additive manufacturing process parameters, mathematical modeling, and optimization. J Manuf Processes. 2023;85:774–782.

[46] Penggang Z, Donglei Y, Xinglong L, et al. Comparison of propane recovery parameter optimization based on the BBD method and CCD method in response surface methodology. J Beijing Univ Chem Technol (Nat Sci Ed). 2024;51(6):16–27.

[47] Jinmin S. Study on the forming efficiency, precision, and mechanical properties of 3d-printed PLA specimens based on response surface analysis. Taiyuan University of Science and Technology; 2024.

[48] Patil S, Sathish T, Makki E, et al. Experimental study on mechanical properties of FDM 3D printed polylactic acid fabricated parts using response surface methodology. AIP Adv. 2024;14(3):035125. doi: 10.1063/5.0191017

[49] Shilin W, Jiawei Z, Peng Z, et al. Research progress on printing strength and forming rate based on FDM forming technology. China Plastics. 2025;39(1):112–117.

[50] Ma Y, Pharr M, Wang L, et al. Soft elastomers with ionic liquid‐filled cavities as strain isolating substrates for wearable electronics. Small. 2017;13(9):1602954. 10.1002/smll.201602954 PMC5332287 28026109

[51] Ma Q, Cheng H, Jang KI, et al. A nonlinear mechanics model of bio-inspired hierarchical lattice materials consisting of horseshoe microstructures. J Mech Phys Solids. 2016;90:179–202. doi: 10.1016/j.jmps.2016.02.012 27087704 PMC4831080

[52] Torabi AR, Shahbaz S, Ayatollahi MR. Tensile fracture prediction of 3d-printed v-notched PLA specimens: application of VIMC-MEMC in conjunction with brittle fracture criteria. Eng Fract Mech. 2024;310:110497. doi: 10.1016/j.engfracmech.2024.110497

[53] Benié K, Barrière T, Placet V, et al. Introducing a new optimization parameter based on diffusion, coalescence and crystallization to maximize the tensile properties of additive manufacturing parts. Addit Manuf. 2023;69:103538. doi: 10.1016/j.addma.2023.103538

[54] Ma Y, Feng X, Rogers JA, et al. Design and application of ‘j-shaped’ stress–strain behavior in stretchable electronics: a review. Lab Chip. 2017;17(10):1689–1704. doi: 10.1039/C7LC00289K 28470286 PMC5505255

[55] Jungebluth P, Haag JC, Sjöqvist S, et al. Tracheal tissue engineering in rats. Nat Protoc. 2014;9(9):2164–2179. doi: 10.1038/nprot.2014.149 25122525

[56] Zengguang L. Study on the enhancement of interface microwave remelting in FDM additive manufacturing of PLA-based parts. China University of Mining and Technology; 2021.

[57] Lin C, Liu L, Liu Y, et al. 4D printing of shape memory polybutylene succinate/polylactic acid (PBS/PLA) and its potential applications. Composite Struct. 2022;279:114729.

[58] Wang S, Li Z, Wang Y, et al. Transcatheter closure of perimembranous ventricular septal defect using a novel fully bioabsorbable occluder: multicenter randomized controlled trial. Sci Bull. 2023;68(10):1051–1059. doi: 10.1016/j.scib.2023.04.027 37179234

[59] Wang Y, Zhu Q, Chen Z, et al. Self-adaptive covalent coating for vascular stents: coordinated coagulation-inflammation regulation to support re-endothelialization for atherosclerosis control. Biomaterials. 2025;325:2026. doi: 10.1016/j.biomaterials.2025.123600 40753783

